# Sex Differences in Outcomes After Endovascular Abdominal Aortic Aneurysm Repair: A Systematic Review and Narrative Synthesis

**DOI:** 10.31083/RCM47920

**Published:** 2026-03-19

**Authors:** Antonio Marzano, Federico Flora, Jihad Jabbour, Ombretta Martinelli, Simone Cuozzo

**Affiliations:** ^1^Department of General and Specialized Surgery and Anesthesiology, Vascular Surgery Unit, “Sapienza” University of Rome, 00161 Rome, Italy; ^2^Heart and Vascular Department, Vascular Surgery Unit, A.O.R.N. “San Giuseppe Moscati”, 83100 Avellino, Italy; ^3^Department of Cardiovascular Sciences, Unit of Vascular Surgery, Fondazione Policlinico Universitario A. Gemelli IRCCS, 00168 Rome, Italy

**Keywords:** endovascular aneurysm repair (EVAR), sex differences, gender disparities, perioperative outcomes, narrative synthesis

## Abstract

**Background:**

Abdominal aortic aneurysm (AAA) is less prevalent in women, yet rupture occurs owing to smaller diameters, leading to higher mortality rates; moreover, higher mortality rates also occur in women after aneurysm repair procedures. Meanwhile, whether women derive comparable benefit from endovascular aneurysm repair (EVAR) remains uncertain, partly because of anatomical constraints, such as smaller-caliber access vessels and more angulated proximal necks. This review evaluates sex-specific perioperative and long-term outcomes after EVAR.

**Methods:**

This study was conducted as a systematic review with narrative synthesis, following the Preferred Reporting Items for Systematic Reviews and Meta-Analyses (PRISMA) 2020 framework. A comprehensive search was conducted in the PubMed/MEDLINE and Scopus databases for studies published between January 2000 and September 2025. Search strings combined controlled vocabulary and free-text terms for “abdominal aortic aneurysm”, “endovascular aneurysm repair”, and “sex” or “gender” or “female”. A predefined Population, Intervention, Comparison, Outcome (PICO) model was used to guide study selection. Comparative observational cohorts, registry or claims analyses, and EVAR-focused meta-analyses reporting sex-stratified outcomes were eligible. Articles were restricted to English. Outcomes included perioperative mortality, major complications, reintervention, and long-term survival. Given the heterogeneity and the availability of recent pooled analyses, quantitative synthesis favored adjusted estimates from high-quality meta-analyses and registries, and no new pooled meta-analysis was performed to avoid data duplication.

**Results:**

A total of 15 studies met the inclusion criteria, encompassing more than 500,000 EVAR procedures. Women consistently exhibited higher early mortality and morbidity after standard infrarenal EVAR. The largest EVAR-focused meta-analysis reported an odds ratio (OR) for 30-day mortality of 1.73 (95% confidence interval (CI) 1.32–2.26) and in-hospital mortality OR of 1.90 (1.43–2.53) for women versus men, with increased risks of limb ischemia (~2.4-fold), renal (OR ~1.7), and cardiac complications (OR ~1.7). Long-term all-cause mortality was higher in women (hazard ratio (HR) 1.23, 95% CI 1.09–1.38). Contemporary registry data indicated similar adjusted mortality but persistently greater access-related morbidity in women, including higher rates of limb ischemia (5.3% vs. 3.2%) and major bleeding (22.0% vs. 15.9%). Perioperative mortality and complications were approximately two-fold higher in women following complex EVAR, defined as fenestrated and/or branched endovascular repair (F/BEVAR) for juxtarenal, pararenal, suprarenal, or thoracoabdominal aneurysms. Additionally, survival remained inferior in those with a ruptured AAA (8-year survival: 36.7% vs. 49.5%).

**Conclusions:**

Women undergoing EVAR continue to experience higher perioperative morbidity and less favorable long-term outcomes compared with men, despite advances in device technology and perioperative care. These disparities largely reflect anatomical and physiological differences, delayed presentation, and underrepresentation in clinical trials and registries. This systematic review and narrative synthesis clarifies sex-specific differences in outcomes after standard infrarenal EVAR and complex F/BEVAR, integrating evidence from contemporary device eras. Sex-aware imaging, individualized access planning, and device design tailored to smaller anatomy are critical to achieving equitable outcomes in endovascular aortic repair.

## 1. Introduction

Abdominal aortic aneurysm (AAA) is more common in men, yet women with AAA typically present at older ages, rupture at smaller diameters, and experience higher rupture-related mortality [1, 2]. Endovascular aneurysm repair (EVAR) has become the preferred treatment for anatomically suitable infrarenal AAA over the past two decades [3], with expanding adoption of fenestrated and branched devices for more complex anatomy [4, 5]. However, whether women derive comparable benefits from EVAR remains uncertain. Multiple studies and meta-analyses have shown that women experience higher perioperative mortality, limb ischemia, renal and cardiac complications, and inferior long-term survival after EVAR compared with men [2, 6, 7].

Sex-related anatomical and physiological differences likely contribute to these disparities. Women generally have smaller-caliber iliofemoral arteries, greater vessel tortuosity, and more angulated or hostile proximal necks, which increase the risk of access injury, bleeding, and endograft-related complications [4, 7, 8]. Women also tend to present later in life and with more advanced disease, largely because screening programmes historically target men and overall awareness among both clinicians and women remains low. Although current guidelines already recommend a lower intervention threshold for women (50 mm) in infrarenal AAA, this adjustment does not fully compensate for sex-based anatomical differences, and evidence does not support repair below established thresholds. For thoracic and thoraco-abdominal aneurysms, similar thresholds are applied to both sexes, although the supporting data remain limited. Body-size–adjusted indices may theoretically refine risk stratification, but their clinical utility has not yet been demonstrated [1, 3]. These anatomical and systemic factors may reduce EVAR eligibility and worsen procedural outcomes [6, 9].

Although previous reviews have examined sex differences in AAA repair, most have combined EVAR and open repair or relied on older device-era data, thereby obscuring EVAR-specific disparities [2]. Women also remain substantially underrepresented in major vascular registries and clinical trials, limiting the precision and generalisability of sex-stratified outcome estimates [8, 10]. With increasing emphasis on personalised aortic care and guideline recommendations highlighting the importance of sex-specific considerations [3], an updated synthesis of sex-based outcomes after contemporary EVAR is needed.

This systematic review therefore evaluates sex-based differences in perioperative and long-term outcomes after standard infrarenal EVAR, and provides a secondary synthesis of outcomes after complex fenestrated/branched EVAR, integrating evidence published between 2000 and 2025. The aim is to quantify disparities and to identify anatomical and procedural contributors that may guide sex-aware clinical practice and future research.

## 2. Materials and Methods

### 2.1 Protocol and Registration

This systematic review followed the Preferred Reporting Items for Systematic Reviews and Meta-Analyses (PRISMA) 2020 framework. The review protocol, including eligibility criteria, data items, and analysis plans, was defined a priori but was not externally registered.

### 2.2 Review Question and Population, Intervention, Comparison, Outcome (PICO)

The review question was: Do women undergoing EVAR for AAA experience different perioperative and long-term outcomes compared with men?

• Population: Adults undergoing standard infrarenal EVAR or complex fenestrated/branched EVAR for abdominal, juxtarenal, pararenal, suprarenal, or thoraco-abdominal aortic aneurysm, including both elective/intact and ruptured cases.

• Intervention/Exposure: EVAR in women.

• Comparator: EVAR in men (biological sex as reported).

• Outcomes: Perioperative mortality (in-hospital or ≤30-day), major complications (access injury, limb ischemia, bleeding, renal or cardiac events), readmission, long-term mortality, reintervention, and endoleak.

• Study designs: Comparative observational cohorts, registry or claims analyses, and EVAR-focused meta-analyses reporting sex-stratified outcomes.

### 2.3 Data Sources and Search Strategy

A comprehensive search was conducted in PubMed/MEDLINE and Scopus for studies published between January 2000 and September 2025, with the final search performed on 30 September 2025. Search strings combined controlled vocabulary (e.g., MeSH terms) and free-text terms for “abdominal aortic aneurysm”, “endovascular aneurysm repair” or “EVAR”, and “sex” or “gender” or “female”. The full search strategies for each database are provided in Table 1.

**Table 1.** **Detailed search strategies used in the electronic databases**.

Database	Platform	Date of last search	Time period covered	Search strategy (exact query)	Limits/Filters	Records retrieved (before de-duplication)
PubMed/MEDLINE	NCBI	30 September 2025	1 January 2000–30 September 2025	Line 1 – Abdominal aortic aneurysm *(“Aortic Aneurysm, Abdominal”[Mesh] OR “abdominal aortic aneurysm”[tiab] OR “abdominal aneurysm”[tiab] OR AAA[tiab])*	- Humans - Adults (≥18 years) - English language - Publication date 2000/01/01–2025/09/30 - Article types: clinical study, cohort, comparative, registry, observational, meta-analysis	n = 153
				Line 2 – Endovascular repair *(“Endovascular Procedures”[Mesh] OR “Endovascular Aneurysm Repair”[Mesh] OR “endovascular aneurysm repair”[tiab] OR EVAR[tiab] OR “endovascular repair”[tiab] OR “stent graft”[tiab])*		
				Line 3 – Sex/gender *(“Sex Factors”[Mesh] OR “Sex Characteristics”[Mesh] OR sex[tiab] OR gender[tiab] OR female[tiab] OR females[tiab] OR woman[tiab] OR women[tiab])*		
				Line 4 – Combine		
				*1 AND 2 AND 3*		
Scopus	Elsevier	30 September 2025	1 January 2000–30 September 2025	*TITLE-ABS-KEY ((“abdominal aortic aneurysm” OR “abdominal aneurysm” OR AAA) AND (“endovascular aneurysm repair” OR “endovascular aortic repair” OR EVAR OR “stent graft” OR “endovascular repair”) AND (sex OR gender OR female OR woman OR women))*	- Document type: Article - Subject area: Medicine - Language: English - Publication years: 2000–2025	n = 117

**Fig. 1.**
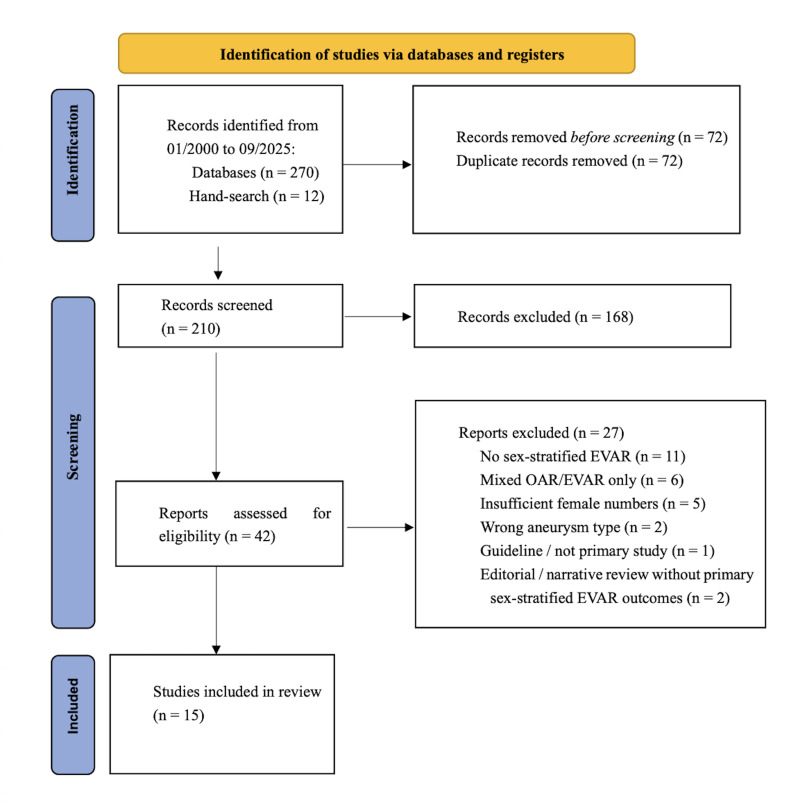
**PRISMA 2020 flow diagram**.

The combined electronic search retrieved 270 records (153 from PubMed/MEDLINE and 117 from Scopus). After removal of 72 duplicates, 198 unique records remained from databases, plus 12 additional records identified by hand-searching, as detailed in the Preferred Reporting Items for Systematic Reviews and Meta-Analyses (PRISMA) 2020 flow diagram (Fig. 1). AAA, abdominal aortic aneurysm; EVAR, endovascular aneurysm repair.

Reference lists of eligible studies and key reviews were manually screened to identify additional records. In addition, the tables of contents of high-volume vascular journals (Journal of Vascular Surgery, European Journal of Vascular and Endovascular Surgery, Annals of Vascular Surgery) were hand-searched to capture in-press or non-indexed studies. These journals were not used as primary data sources but served to complement the database search. Articles were limited to those published in English.

### 2.4 Eligibility Criteria

Studies were included if they met the following criteria:

• Population: Adults undergoing EVAR for infrarenal or complex abdominal aneurysms, as defined by the European Society for Vascular Surgery (ESVS) guidelines [3] or thoraco-abdominal aortic aneurysm, including both elective/intact and ruptured cases. Isolated thoracic aneurysm repairs without an abdominal component were excluded.

• Comparator: Female versus male sex (biological sex as reported).

• Outcomes: At least one of the following outcomes reported by sex, perioperative mortality (in-hospital or ≤30-day), major complications (access injury, limb ischemia, bleeding, renal or cardiac events), readmission, long-term mortality, reintervention, or endoleak.

• Study design: Comparative observational cohorts, registry or claims analyses, and meta-analyses reporting EVAR-specific, sex-stratified outcomes.

Exclusion criteria were:

• Studies lacking sex-specific EVAR data.

• Mixed open/EVAR results without separate reporting for EVAR.

• Isolated thoracic aneurysms without abdominal involvement.

• Very small studies (total sample <50 or <20 women), which were unlikely to provide stable sex-stratified estimates.

• Non-human or paediatric studies.

• Non-English publications.

Bailout techniques such as chimney EVAR and physician-modified endografts were not grouped with complex EVAR but are described qualitatively when relevant.

### 2.5 Study Selection, Data Extraction, and Risk of Bias Assessment

Two reviewers independently screened titles and abstracts, followed by full-text assessment for eligibility. Study-level data were extracted using a standardized template including study design, setting, sample size by sex, indication (intact or ruptured), aneurysm extent (infrarenal vs juxtarenal/pararenal/suprarenal vs thoraco-abdominal), type of repair (standard EVAR vs F/BEVAR), follow-up duration, and adjusted outcome estimates. Disagreements were resolved by consensus.

Risk of bias in observational studies was assessed using the Risk Of Bias In Non-randomized Studies of Interventions (ROBINS-I) tool, and the methodological quality of meta-analyses was appraised with AMSTAR-2 (A MeaSurement Tool to Assess Systematic Reviews). Quality judgments were summarized descriptively rather than used for quantitative weighting, given the heterogeneity of study designs and populations.

### 2.6 Data Synthesis and Analysis

Data were synthesized across four main outcome domains: perioperative mortality and morbidity, long-term survival, reintervention, and anatomical or procedural differences. Quantitative results were extracted as adjusted odds ratios (ORs) or hazard ratios (HRs) for women versus men, prioritizing multivariable or propensity-score matched estimates when available.

Where pooled results existed from high-quality EVAR-focused meta-analyses, those estimates were cited directly as summary statistics rather than recalculated. Single-cohort or registry studies were narratively compared with meta-analytic benchmarks to assess consistency of findings.

Heterogeneity was qualitatively assessed across study designs, device generations, and populations (single-centre vs national datasets). In view of methodological variability, overlapping patient populations, and the presence of robust recent meta-analyses, a new pooled meta-analysis was not performed. Instead, this review was structured as a systematic review with narrative synthesis. Results are presented descriptively in Tables 2,3,4 (Ref. [1, 2, 4, 5, 6, 7, 8, 9, 11, 12, 13, 14, 15, 16, 17]; Ref. [6, 7, 11, 15]; Ref. [1, 8]) and summarized in the PRISMA 2020 flow diagram (Fig. 1). Statistical measures are expressed as reported in the original sources, with 95% confidence intervals where available.

**Table 2.** **Included studies and main characteristics**.

First author	Year	Design/Data source	Setting	Key female vs male findings	Adjusted effect (if reported)
Liu [6]	2020	Meta-analysis (36 cohorts)	Elective EVAR	Women ↑ 30-day & in-hospital mortality; ↑ limb ischemia, renal & cardiac complications; ↑ long-term mortality	30-d aOR 1.73 (1.32–2.26); in-hospital OR 1.90 (1.43–2.53); long-term HR 1.23 (1.09–1.38)
Pouncey [2]	2021	Meta-analysis (EVAR & OAR; EVAR subgroup)	Elective EVAR	Women ↑ 30-day mortality; ↑ arterial injury/limb ischemia; ↑ renal & cardiac complications	EVAR 30-d OR ∼1.86
Behrendt [8]	2021	Nationwide claims (Propensity Score-matched)	Elective EVAR	Similar adjusted mortality; Women ↑ acute limb ischemia (5.3% vs 3.2%) & major bleeding (22.0% vs 15.9%)	HR mortality 0.91 (0.76–1.08)
Erben [9]	2021	Multicenter cohort	Elective EVAR	Similar in-hospital & 3-y mortality; Women ↑ return to Open Repair, ICU, rehab discharge	-
O’Donnell [4]	2020	Device registry	Elective EVAR	Comparable 5-y survival (HR 0.89, 0.61–1.29); Women ↑ type IA endoleak (10% vs 1%)	-
Corsi [7]	2022	Single-center	Elective EVAR	Women ↓ 5-y survival; ↑ 1- & 5-y reintervention	5-y mortality HR 1.8 (1.1–2.9); reintervention HR 2.4 (1.1–4.9)
Lo [1]	2014	Regional registry	Elective EVAR & ruptured AAA	Women older; smaller diameters; ↓ EVAR use for intact; ↑ arterial injury	-
Li [15]	2022	Nationwide cohort	Ruptured AAA (EVAR & OAR)	Women ↑ periop & 8-y mortality after rAAA repair	-
de Guerre [11]	2020	ACS-NSQIP targeted	Complex EVAR	Women ↑ perioperative mortality & major complications; disparity not seen after complex OAR	Mortality OR ∼2.5; complications OR ∼2.0
Behrendt [5]	2021	Single-centre complex EVAR	Complex EVAR	Women ↑ in-hospital mortality & complications (incl. SCI)	-
Jin [13]	2023	Systematic review/meta-analysis	Complex EVAR	Female sex ↑ mortality & complications after complex EVAR	-
Gormley [14]	2024	National registry	Ruptured AAA (EVAR & OAR)	Women ↑ early EVAR mortality (25.9% vs 18.9%)	-
Tumer [16]	2021	Single-center (Turkey)	Elective EVAR	Comparable mid-term mortality & reintervention between sexes	-
Varkevisser [12]	2020	Device registry (Ovation graft)	Elective EVAR	Similar 5-y outcomes; validates feasibility in women	-
Forbes [17]	2023	Multicentre F/BEVAR outcomes	Complex EVAR	Women ↑ early morbidity after F/BEVAR	-

OAR, open aortic repair; ICU, intensive care unit; SCI, spinal cord ischemia; ACS-NSQIP, American College of Surgeons National Surgical Quality Improvement Program; IFU, instruction for use; OR, odds ratio; HR, hazard ratio; F/BEVAR, Fenestrated/Branched Endovascular abdominal aortic repair; aOR, adjusted odds ratio; rAAA, ruptured abdominal aneurysm aortic; y, years.

**Table 3.** **Key adjusted effect estimates by outcome (women vs men)**.

Outcome	Best available adjusted estimate	Source
30-day mortality (intact EVAR)	OR 1.73 (CI 1.32–2.26)	Liu [6]
In-hospital mortality (intact EVAR)	OR 1.90 (CI 1.43–2.53)	Liu [6]
Limb ischemia	OR ∼2.44	Liu [6]
Renal complications	OR 1.73 (CI 1.32–2.27)	Liu [6]
Cardiac complications	OR 1.68 (CI 1.22–2.33)	Liu [6]
Long-term all-cause mortality	HR 1.23 (CI 1.09–1.38)	Liu [6]
Complex EVAR: perioperative mortality	OR ∼2.5	de Guerre [11]
Complex EVAR: major complications	OR ∼2.0	de Guerre [11]
Elective EVAR 5-y survival	HR 1.80 (CI 1.10–2.90) unfavorable for women	Corsi [7]
Elective EVAR 5-y reintervention	HR 2.40 (CI 1.10–4.90) higher in women	Corsi [7]
rAAA repair: 8-y survival	Lower in women (survival 36.7% ♀ vs 49.5% ♂)	Li [15]

CI, confidence interval.

**Table 4.** **Anatomical/presentation differences and treatment patterns**.

Feature	Women	Men	Source
Age at intact AAA repair (median)	75 y	72 y	Lo [1]
Aneurysm diameter at intact repair (median)	57 mm	59 mm	Lo [1]
EVAR use in intact AAA (%)	50%	60%	Lo [1]
Access/arterial injury (%)	5.4%	2.7%	Lo [1]
Acute limb ischemia after elective EVAR (%)	5.3%	3.2%	Behrendt [8]
Major bleeding after elective EVAR (%)	22.0%	15.9%	Behrendt [8]

When multiple adjusted estimates existed for the same outcome, we selected the most methodologically robust estimate according to a predefined hierarchy: (1) multivariable or propensity-adjusted models; (2) larger datasets or national registries; (3) contemporary device-era studies; and (4) pooled adjusted estimates from prior meta-analyses when these did not introduce duplication.

## 3. Results

### 3.1 Evidence Base

Fifteen studies met the inclusion criteria: two EVAR-focused meta-analyses, six national registry or claims analyses, and seven single-centre or multicentre observational cohorts reporting sex-stratified EVAR outcomes. Together, these studies represent more than 500,000 EVAR procedures, predominantly derived from large registries and pooled data (Table 2). Study designs, populations, and key findings are summarized in Tables 2,3,4.

The primary focus of this review is standard infrarenal EVAR. Complex EVAR (F/BEVAR) and ruptured AAA repairs are presented in dedicated subsections to avoid conflation with standard elective infrarenal procedures.

### 3.2 Quantitative Summary

Across pooled and adjusted analyses, women experienced higher perioperative mortality and morbidity after EVAR compared with men.

In the largest EVAR-focused meta-analysis [6], women had 30-day mortality OR 1.73 (95% CI 1.32–2.26), in-hospital mortality OR 1.90 (1.43–2.53), and higher risks of limb ischemia (OR ~2.4), renal complications (OR ~1.7), and cardiac complications (OR ~1.7). Long-term all-cause mortality was also higher in women (HR 1.23 [1.09–1.38]) (Table 3).

In registry-based cohorts, adjusted mortality was often similar between sexes, but women consistently showed higher access-related morbidity, for example, in German claims data [8]: acute limb ischemia 5.3% vs 3.2% and major bleeding 22.0% vs 15.9% (women vs men).

For complex EVAR (fenestrated/branched/pararenal), multiple studies reported markedly higher perioperative mortality and major complications in women, with OR ~2.5 for mortality and ~2.0 for major complications [11]. For ruptured AAA, women had persistently higher perioperative and late mortality following both EVAR and open aortic repair (OAR). In the large national cohort by Li *et al*. [15], 8-year survival was 36.7% in women versus 49.5% in men.

### 3.3 Narrative Synthesis

#### 3.3.1 Perioperative Risk

Previously published pooled estimates from existing meta-analyses indicate that women experience higher early mortality and morbidity after standard EVAR, primarily due to access-related complications (arterial injury, limb ischemia) and higher rates of cardiorenal events [2, 6, 8, 9]. These differences appear across device generations, though some recent cohorts suggest attenuation of mortality differences with improved perioperative care and patient selection [4].

#### 3.3.2 Adjusted Mortality in Real-World Cohorts

Several large datasets demonstrated comparable adjusted perioperative mortality between sexes despite higher complication rates in women, suggesting that improved selection and perioperative optimization may have mitigated sex-related differences in death, while morbidity disparities remain [4, 8, 9]. However, increased early complications may still contribute to excess long-term mortality in some series [6, 7].

#### 3.3.3 Long-Term Outcomes

Meta-analytic data confirm higher all-cause mortality in women after EVAR [6]. Single-centre studies such as Corsi *et al*. [7] showed increased 5-year reintervention (HR 2.4 [1.1–4.9]) and mortality (HR 1.8 [1.1–2.9]) in women. Conversely, device registries such as ENGAGE and Ovation demonstrated broadly similar survival between sexes but differing patterns of endoleak, particularly type IA endoleak, suggesting an important role of anatomical and device-specific factors [4, 12].

#### 3.3.4 Complex EVAR

In complex EVAR (fenestrated/branched), multiple analyses [5, 10, 11, 13] showed approximately twofold higher perioperative mortality and complication rates in women, including rare events such as spinal cord ischemia. Women undergoing F/BEVAR more often have smaller-caliber target vessels and more challenging aortic and branch anatomy, which may increase the risk of technical failure, end-organ ischaemia, and early complications.

#### 3.3.5 Ruptured AAA

For ruptured AAA, national and multicentre datasets consistently demonstrated worse perioperative and long-term survival for women, with early EVAR mortality of 25.9% vs 18.9% in a large international registry [14] and inferior 8-year survival in national cohort data [15]. These findings suggest that sex-based disparities persist even when emergent EVAR is used.

#### 3.3.6 Anatomical and Procedural Contributors

Sex differences in EVAR outcomes are strongly linked to anatomy and procedural constraints. Women have smaller iliofemoral arteries, more tortuous vessels, and more angulated or conical proximal necks [1, 4, 7, 9], and are more likely to undergo EVAR at or beyond device instructions for use [2, 6]. These features complicate device delivery and fixation, increasing the risk of access injury, bleeding, and endograft failure [8, 9]. Older age, higher comorbidity burden, and underuse of screening and body-size–adjusted thresholds compound these anatomical challenges [1, 3, 10]. These findings support guideline calls for sex-aware imaging, access planning, and surveillance strategies [3].

## 4. Discussion

This systematic review and narrative synthesis summarizes two decades of evidence (2000–2025) on sex-based differences in outcomes after endovascular abdominal aortic aneurysm repair. Across pooled analyses, registry datasets, and single-centre studies, women consistently demonstrated higher perioperative mortality and morbidity compared with men. The disparity is most evident for access-related complications, cardiorenal events, and complex or ruptured aneurysm presentations. Although several recent large cohorts have reported similar adjusted mortality between sexes, the persistence of excess morbidity in women indicates that sex-based anatomical and physiological differences remain influential determinants of outcome.

Our findings align with and extend previous systematic reviews. The meta-analysis by Liu *et al*. [6] established that female sex was independently associated with higher 30-day and in-hospital mortality following EVAR, together with increased limb ischemia and renal or cardiac complications. Pouncey *et al*. [2] similarly found higher short-term mortality and access-related injury among women, although their analysis combined open and endovascular repairs.

The persistent nature of sex-based disparities despite advances in peri-operative care and device technology has also been underscored by Pouncey *et al*. [18] in a subsequent temporal-trend analysis (2022), which confirmed that the ‘gender gap’ in AAA repair outcomes has not been fully closed.

By focusing specifically on EVAR and complex F/BEVAR and including more contemporary studies, the present review confirms that these differences persist into the current device era despite advancements in stent-graft design, imaging, and operator experience.

The mechanisms underlying these disparities are multifactorial. Anatomically, women have smaller-caliber iliofemoral arteries, greater iliac tortuosity, and more acutely angulated or conical proximal necks. These features complicate device delivery and fixation, increasing the risk of access injury, bleeding, endoleak, and device failure. Moreover, women often present at older ages with a higher comorbidity burden, partly due to screening programmes historically targeting men and intervention thresholds based on absolute aneurysm diameter rather than aortic size index [1]. Systematic under-recognition and delayed referral likely contribute to higher perioperative risk and reduced long-term survival.

While technological progress and improved perioperative care have narrowed the mortality gap, they have not eliminated sex disparities in morbidity. Registry and claims data [1, 4, 8, 12, 14] suggest that women experience more bleeding and access-related complications even when adjusted for confounders, underscoring the importance of proactive preoperative access assessment and selective use of surgical or endovascular conduits. In complex EVAR [5, 11, 13, 17], women continue to exhibit approximately twofold higher perioperative mortality and major complication rates, reflecting the cumulative effect of challenging anatomy, limited device compatibility, and older age. For ruptured aneurysms [14, 15], women remain disadvantaged, with early mortality often exceeding 25% despite endovascular treatment and long-term survival remains inferior.

Beyond infrarenal and pararenal EVAR, emerging evidence from thoraco-abdominal and inner branch endovascular repair indicates that endovascular approaches can achieve acceptable results even in extensive aneurysms [19, 20]. However, sex-specific outcomes have not yet been explored in this setting. Understanding whether the sex-based disparities observed in infrarenal EVAR also extend to thoraco-abdominal repairs remains an important research need.

In addition, frailty has emerged as an independent determinant of perioperative and long-term outcomes after complex EVAR [21, 22]. Frailty captures physiologic vulnerability beyond chronological age and anatomical complexity, yet current literature rarely stratifies its impact by sex. Future studies should evaluate whether frailty exerts differential prognostic effects in women versus men, given the interaction between biological sex, comorbidity, and recovery potential.

These findings have important clinical implications. They reinforce the need for sex-aware imaging, planning, and surveillance strategies, including careful evaluation of iliofemoral access and proximal neck configuration before EVAR, and consideration of individualized thresholds for intervention based on aortic size index. Device manufacturers should prioritize low-profile delivery systems and designs optimized for smaller access vessels. In research, systematic sex- and frailty-stratified reporting should become standard, with adequate female representation in registries and trials to improve the validity of risk prediction models. Moreover, building on the experience of international, web-based initiatives such as the RIVAS survey [23], future research should include a dedicated, multicentre survey among vascular surgeons to assess awareness of sex-based differences in aortic diseases and to explore how perceived gender-related risk modifies clinical decision-making, patient selection, and procedural strategy in contemporary aortic practice.

Despite inherent limitations, mainly the predominance of observational designs, heterogeneous adjustment, and limited female representation, the consistency of findings across independent cohorts supports the robustness of these conclusions.

In summary, women undergoing EVAR experience higher early morbidity and often worse long-term outcomes compared with men, even in contemporary practice. Whether similar disparities exist in thoraco-abdominal endovascular repair remains to be established. Integrating sex and frailty assessment into patient selection, device development, and follow-up strategies will be essential steps toward genuinely personalized aortic care.

### 4.1 Clinical Implications

**(1) Pre-EVAR planning in women:** mandatory access assessment (computed tomography angiography with ilio-femoral diameters and tortuosity), a low threshold for surgical or endovascular conduits or alternative access, and proactive renal and cardiac protection strategies.

**(2) Intra-operative strategy:** anticipate adjunctive iliac techniques; consider sheath profiles and delivery angles mindful of smaller vessels; meticulous haemostasis given higher bleeding risk.

**(3) Post-EVAR surveillance:** equal or more vigilant early imaging for limb compromise; structured late surveillance acknowledging women’s higher all-cause mortality risk.

**(4) Complex EVAR counselling:** discuss higher perioperative risks for women; consider whether open repair may equalize risk in select anatomies.

**(5) Policy & research:** routine sex-stratified reporting; incorporate aortic size index and access anatomy into indications and risk models; ensure device trials enrol adequate numbers of women.

### 4.2 Strengths and Limitations

Strengths of this review include the strict EVAR and complex EVAR focus; inclusion of the most informative meta-analyses and large, contemporary cohorts; and a structured quantitative and narrative summary of sex-specific effects. Limitations include the predominance of observational data with residual confounding; heterogeneity in device eras and adherence to instructions for use; limited female sample sizes in some studies; variability in endoleak and reintervention reporting; and restriction to English-language publications. In addition, although multiple databases were searched and hand-searching was performed, some relevant studies may have been missed.

## 5. Conclusions

Over the last two decades, the body of evidence consistently demonstrates that women undergoing EVAR repair remain at higher risk of perioperative complications and often experience inferior long-term outcomes compared with men. Although advances in imaging, device design, and operator experience have narrowed mortality gaps in some settings, anatomical, biological, and systemic factors continue to drive outcome disparities.

These differences extend beyond infrarenal repair, raising important questions for complex [24] and thoraco-abdominal endovascular procedures, where early results appear encouraging but sex-specific outcomes remain largely unexplored, despite reported cases in the literature of highly complex endovascular procedures performed in women [25]. Moreover, the growing recognition of frailty as a determinant of prognosis suggests that future analyses should stratify outcomes by both sex and frailty status, to capture the true interplay between vascular anatomy, physiological reserve, and procedural risk.

Achieving equity in aortic care will require concerted efforts at multiple levels: refining anatomical thresholds and device instructions for use to account for sex-based variation, ensuring balanced recruitment in clinical trials, and standardizing sex-stratified and frailty-adjusted reporting in registries and outcome studies. Ultimately, a personalized approach to EVAR, integrating sex, anatomy, frailty, and comorbidity, offers the greatest potential to improve both short- and long-term results. Future device innovation and clinical research should explicitly address these biological and structural determinants to advance truly inclusive, evidence-based aortic repair.

## Availability of Data and Materials

All data supporting the findings of this systematic review are available within the article and its supplementary materials. The datasets analyzed consist exclusively of previously published studies cited in the References section.

## Supplementary Material

Supplementary material associated with this article can be found, in the online version, at https://doi.org/10.31083/RCM47920.
